# Maternal perinatal depressive disorders and the risk of attention deficit and hyperactivity disorder in offspring: A retrospective cohort study using linked data

**DOI:** 10.1192/j.eurpsy.2025.1181

**Published:** 2025-08-26

**Authors:** B. S. Tusa, R. Alati, K. Betts, G. Ayano, B. Dachew

**Affiliations:** 1 School of Population Health, Curtin University, Perth, Australia; 2 Department of Epidemiology and Biostatistics, College of Health and Medical Sciences, Haramaya University, Haramaya, Ethiopia; 3 Institute for Social Science Research, The University of Queensland, Brisbane; 4 enAble Institute, Curtin University, Perth, Australia

## Abstract

**Introduction:**

Maternal perinatal depression may increase the risk of neurodevelopmental disorders such as attention deficit hyperactivity disorder (ADHD), in children, either directly or through indirect pathways involving adverse birth outcomes.

**Objectives:**

This study assesses the risk of ADHD in offspring born to mothers with perinatal depressive disorders, examining both the direct and indirect pathways through adverse birth outcomes such as low birth weight, low APGAR scores, and preterm birth.

**Methods:**

The study employed a retrospective cohort design, utilising administrative-linked health data from New South Wales. Maternal perinatal depressive disorders and offspring ADHD were identified using the International Classification of Diseases (ICD-10) codes. A generalised linear model with a binomial distribution and a log link function was applied to estimate the direct association. Additionally, a mediation analysis examined the mediational effect of low birth weight, low APGAR scores, and preterm birth on the association between maternal antenatal depressive disorder and ADHD.

**Results:**

After adjusting for potential confounders, offspring of mothers with antenatal, postnatal, and perinatal depressive disorders are respectively 2.10 times (RR = 2.10, 95% CI = 1.75–2.53), 1.81 times (RR = 1.81, 95% CI = 1.41–2.31), and 2.16 times (RR = 2.16, 95% CI = 1.84–2.54) more likely to have ADHD compared to their counterparts. The impact of maternal antenatal depressive disorder on offspring ADHD was mediated by preterm birth, but not by low birth weight or low APGAR scores. The proportion of the total effect mediated by preterm birth was only 0.73%, indicating this mediation effect was very minimal, about 45 times smaller than the direct effect.

**Conclusions:**

Our study revealed that maternal perinatal depressive disorders are associated with an increased risk of offspring ADHD, with very minimal or no mediating effects from adverse birth outcomes. Therefore, implementing early intervention strategies aimed at improving maternal mental health is crucial to reducing the risk of ADHD in children.

**Disclosure of Interest:**

None Declared

